# Prescription of Blood Lymphocyte Immunophenotyping in the Diagnosis of Lymphoid Neoplasms in Older Adults

**DOI:** 10.3390/jcm11061748

**Published:** 2022-03-21

**Authors:** Jérémie Vovelle, Céline Row, Fabrice Larosa, Julien Guy, Anca-Maria Mihai, Marc Maynadié, Jérémy Barben, Patrick Manckoundia

**Affiliations:** 1 “Pôle Personnes Âgées”, Hospital of Champmaillot, University Hospital, 21079 Dijon, France; jeremie.vovelle@chu-dijon.fr (J.V.); fabrice.larosa@chu-dijon.fr (F.L.); anca-maria.mihai@chu-dijon.fr (A.-M.M.); jeremy.barben@chu-dijon.fr (J.B.); 2 Department of Biological Hematology, University Hospital, 21079 Dijon, France; celine.row@chu-dijon.fr (C.R.); julien.guy@chu-dijon.fr (J.G.); marc.maynadie@chu-dijon.fr (M.M.); 3 INSERM U-1093, Cognition, Action and Sensorimotor Plasticity, University of Burgundy Franche-Comté, 21000 Dijon, France

**Keywords:** aged 75 and over, blood lymphocyte immunophenotyping, lymphoid neoplasms

## Abstract

Lymphoid neoplasms are a heterogeneous group of lymphoid neoplastic diseases with multiple presentations, and varying prognoses. They are especially frequent in older patients (OPs) and the atypism of this frail elderly population can make the diagnostic process even more difficult. Blood lymphocyte immunophenotyping (BLI) is essential in rapid noninvasive diagnosis orientation and guides complementary investigations. To our knowledge, BLI prescription has never been evaluated in OPs. We hypothesized that, when there is a suspicion of lymphoid neoplasm in the geriatric population, a BLI is performed in view of various clinical or biological abnormalities. This study aimed to: (1) describe the characteristics of hospitalized OPs having undergone BLI for suspected lymphoid neoplasm, (2) identify the causes leading to BLI prescription, and (3) identify the most profitable criteria for BLI prescription. This was a descriptive retrospective study on 151 OPs aged ≥75 years who underwent BLI over a 2-year period. Regarding BLI prescriptions, eight had lymphocytosis, constituting the “lymphocytosis group” (LG+), while the 143 others had BLI prescribed for reasons other than lymphocytosis (LG−), mainly general weakness and anemia. In the LG−, we compared OPs with positive and negative BLI results. The criteria found to be profitable for BLI prescription were lymphadenopathy, splenomegaly, lymphocytosis, and thrombocytopenia. BLI identified circulating lymphoid neoplasms (positive BLI) in 21/151 OPs, mainly marginal zone lymphoma and chronic lymphocytic leukemia. In polymorbid OPs, as per our study population, the diagnostic and therapeutic complexity explained in part the sole use of indirect and minimally invasive diagnostic techniques such as BLI.

## 1. Introduction

Lymphoid neoplasms are a heterogeneous group of lymphoid diseases, which are clonal tumors originating from lymphocytes, B-cells (B-cell lymphoid neoplasms) or T-cells (T-cell lymphoid neoplasms) [1]. Lymphoid neoplasms occur mainly in older adults and their incidence has increased over time [1], particularly for diffuse large B-cell lymphoma (DLBCL). This group of diseases has highly variable clinical (existence of extra-nodal forms) and biological expressions, as well as very different prognoses, from indolent to aggressive depending on the subset [2]. In older patients (OPs), the accumulation of geriatric frailty and various geriatric syndromes leads to more complex disease presentation, resulting in a highly challenging diagnostic process diagnostic process for lymphoid neoplasms [3,4].

In current practice, it is possible to perform blood lymphocyte immunophenotyping (BLI) by multiparameter flow cytometry for detection of small-cell circulating clonal lymphoid proliferation and subset identification in the event of suspected lymphoid neoplasm [5]. BLI is a rapid noninvasive test that provides diagnostic orientation and can guide complementary investigations, such as solid biopsy for histological confirmation. BLI is especially prescribed in patients with lymphocytosis. The Matutes score distinguishes chronic lymphocytic leukemia (CLL) (score ≥ 4) from other B-cell lymphoid neoplasmss [6,7]. However, lymphocytosis and circulating blood phase are not systematic in all lymphoid neoplasms.

We hypothesized that in case of lymphoid neoplasm suspicion in the elderly population, a BLI test is carried out in view of various clinical or biological abnormalities, including lymphadenopathy(ies), splenomegaly, hepatomegaly, general weakness, unexplained fever, formed elements of blood disorders (lymphocytosis, anemia, thrombocytopenia, monocytosis), monoclonal gammapathy and unexplained inflammatory syndrome. To our knowledge, BLI prescription has never been evaluated in OPs.

The main objective of this study was to assess the indications of BLI prescription in a hospitalized geriatric population with suspected lymphoid neoplasm. The second objective was to distinguish possible profitable clinical and/or biological characteristics justifying BLI prescription. 

## 2. Methods

### 2.1. Study Design

This retrospective observational descriptive study was carried out in the two geriatric departments (an acute geriatric care unit and a geriatric rehabilitation unit) of the Dijon-Bourgogne University Hospital.

Data were collected for OPs hospitalized between 1 January 2018 and 31 December 2019.

This study was conducted in accordance with the Declaration of Helsinki and French national standards.

### 2.2. Population

We included all hospitalized OPs (aged ≥ 75 years) who had a BLI prescribed for the first time during their hospital stay. OPs were excluded when BLI was added by the hematology laboratory following morphological abnormalities on the blood smear.

The Ethics Committee of our institution was consulted and approved this retrospective study, which had no effect on patient management.

### 2.3. BLI Technique

BLI was performed by the flow cytometry platform of the biological hematology laboratory of the Dijon-Bourgogne University. Total EDTA blood samples were processed in a “lysis-no wash” protocol within 24 h after blood collection. A first screening panel was used with the nine-color monoclonal antibody combination: Kappa-FITC/Lambda-PE/CD4-PECF594/CD5-PC5.5/CD8-PC7/CD3-APC/CD19-AA750/CD16 BV421/CD45-KO [5]. Positivity for clonal proliferation was determined by light chain restriction. In case of monoclonal B lymphoid population proliferation, three identification tubes were processed in order to distinguish B-cell lymphoid neoplasms subsets: tube 1 (CD81-FITC/CD43-PE/CD38-PECF594/CD5-PC5.5/CD23-PC7/CD79b-APC/CD22-AA700/CD19-AA750/FMC7-PB/CD45-KO), tube 2 (CD103-FITC/CD10-PE/CD13-PE-Dylight/CD5-PC5.5/CD11c-PC7/CD123-APC/CD25-AA700/CD19-AA750/CD20-PB/CD45-KO) and tube 3 (CD180-PE/CD5-PC5.5/CD19-AA750/CD45-KO) [5]. In case of monoclonal T lymphoid population proliferation, two identification tubes were used: tube 1 (HLADR-FITC/CD10-PE/CD4-PECF594/CD5-PC5.5/CD8-PC7/CD2-APC/CD7-AA700/CD25-AA750/CD3-PB/CD45-KO) and tube 2 (TCRGD-FITC/TCRAB-PE/CD4-PECF594/CD5-PC5.5/CD8-PC7/CD2-APC/CD56-AA700/CD16-AA750/CD57-BV421/CD45-KO) [5].

Sample acquisition was performed using a Navios® (Beckman-Coulter, Brea, CA, USA) on at least 5000 lymphocytes. Data were then analyzed using Kaluza® software, version 2.1 (Beckman Coulter, Brea, CA, USA).

BLI was considered positive when it detected and identified a cluster of clonal lymphoid proliferation consistent with lymphoid neoplasm with circulating blood phase. BLI was considered negative when no circulating clone was identified (normal lymphocyte distribution).

### 2.4. Collected Data

The collected epidemiological data included age, sex, and history of lymphoid neoplasm. BLI prescription was justified by the prescribing physician.

The collected clinical data included tumoral syndrome (lymphadenopathy(ies), hepatomegaly, splenomegaly), and general symptoms (clinical B-symptoms).

Collected blood data included leukocyte, lymphocyte, eosinophil, monocyte, hemoglobin, and platelet counts, albuminemia, and C-reactive protein, as well as serum protein electrophoresis and immunofixation (specifying the result: normal, monoclonal gammapathy, polyclonal hypergammaglobulinemia, or hypogammaglobulinemia).

The collected geriatric data were: (1) age-adjusted Charlson comorbidity index (ACCI) [8], (2) number of medications at admission (polypharmacy was defined by the consumption of at least 5 different drugs per day [9]), (3) place of residence, (4) Mini Mental State Examination (MMSE) score, (5) baseline ambulatory status (the OP was considered “independent” if walking without help or with a cane and “dependent” if requiring a walker and/or assistance during transfers or bed-chair confinement, (6) score on the Activity Daily Living (ADL) scale [10], and (7) level of medical intervention [11].

Data for complementary investigations were also collected, such as histology confirmation (solid biopsy) and disease staging by imaging (thoracic–abdominal–pelvic computed tomography, positron emission tomography–computed tomography) and/or myelogram/bone marrow biopsy. 

Finally, data were also collected for follow-up care including hematological and/or oncogeriatric evaluation, and in confirmed lymphoid neoplasm, initiation or not of a specific treatment.

### 2.5. Composition of the Groups

Patients were divided in two groups depending on BLI prescription in the presence or absence of lymphocytosis. The “lymphocytosis group” (LG+) consisted of OPs with BLI prescribed for lymphocytosis ≥ 5 G/L. In the “absence of lymphocytosis group” (LG−), BLI was prescribed for other clinical or biological reasons.

The LG− was divided into two subgroups depending on BLI results: BLI positive, identifying circulating lymphoid neoplasm, or BLI negative, defined by the absence of circulating lymphoid neoplasm.

### 2.6. Statistical Analysis

Qualitative data were expressed as numbers and percentages. Quantitative data were expressed as means with standard deviations and extreme values. The two subgroups of LG− were compared using univariate analysis. The Chi-square test or Fischer test was used for qualitative variables and the Student’s *t*-test for quantitative variables. 

BiostaTGV© (Institut Pierre Louis, Paris, France), a free website, was used for all statistical analyses on Excel®, version 16.16.21 (Microsoft Corporation, Redmond, WA, USA).

Statistical significance was set at *p* ≤ 0.05.

## 3. Results

### 3.1. Included Patients

**Figure 1:**
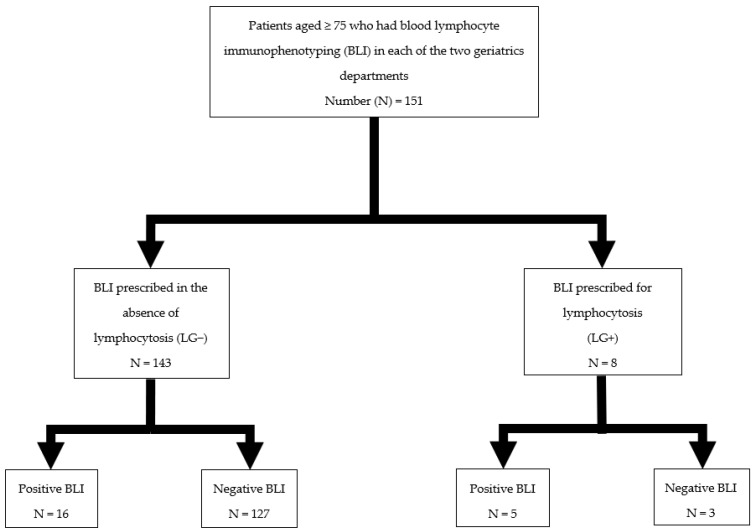
Study flowchart.

A total of 151 OPs had had a BLI prescribed during the study period. Their average age was 86.7 ± 5.7 years (range 75–97) and 51% were men; 8 (5.3%) patients had lymphocytosis (LG+), while 143 (94.7%) did not (LG−) (Figure 1).

The mean ACCI was 6.4 ± 1.7 (range 3–15) and 102 OPs (67%) had an ACCI ≥ 6. The mean ADL score was 3.9 ± 1.6 (range 0–6); 57 OPs (38%) had an ADL score ≥ 5. In addition, 137 of the 151 OPs received a cognitive assessment by MMSE with an average score of 18.7 ± 6.2 (range 3–30).

Finally, 28% of OPs had a level of medical intervention giving them access to intensive care in the event of deterioration.

### 3.2. Characteristics of LG+ 

The LG+ included eight patients aged 85.0 ± 5.3 years, and 87.5% women. 

Five (62.5%) had positive BLI identifying three (60%) CLL and two (40%) circulating phase of marginal zone lymphoma (MZL) (Table 1). Only one MZL patient had histological confirmation of MZL infiltration in bone marrow biopsy. His ACCI, ADL score, and MMSE score were, respectively, 5, 6/6, and 26/30.

Three OPs (37.5%) had negative BLI with neither signs of a tumor nor cytopenia, and lymphocytosis was reactive.

### 3.3. Characteristics of LG− 

#### 3.3.1. Indications of BLI Prescription in LG−

While BLI was prescribed to investigate lymphocytosis in the LG+, the most common reasons for prescribing BLI in the absence of lymphocytosis was general weakness (19.6%) and anemia (10.5%) (Table 2).

#### 3.3.2. Characteristics of LG− Depending on BLI Result

The LG− included 143 patients aged 86.8 ± 5.7 years and was 53.1% male. Most of these patients lived at home (85%), had polypharmacy (78%), and were ambulating independently (54%).

A total of 16 OPs (11.1%) were found to have positive BLI: seven MZL (44%), three monoclonal B-cell lymphocytosis (MBL) (19%), two small lymphocytic lymphoma (SLL) (12.5%), two T-cell lymphoid neoplasms (12.5%), one lymphoplasmacytic lymphoma (LPL) (6%), and one follicular lymphoma (FL) (6%).

Table 3 compares OPs with positive and negative BLI in the LG−.

OPs in the LG− with positive BLI had significantly more comorbidities according to ACCI (*p* = 0.03), and cognitive disorders according to MMSE score (*p* < 0.01). Among the biological markers, albuminemia was significantly higher in this subgroup (*p* = 0.02).

As concerns clinical characteristics, a positive BLI (identifying a circulating lymphoid neoplasm blood phase) was significantly more frequent among OPs with lymphadenopathy(ies) (*p* = 0.05) and splenomegaly (*p* = 0.02).

For complete blood count, LG−/BLI positive patients had significantly lower platelet count and higher lymphocyte count compared to those with negative BLI (*p* < 0.01 for both). However, the presence of strict thrombocytopenia (< 150 G/L) was not significantly associated with positive BLI (*p* = 0.06).

There was no significant difference between the two subgroups for the prescription of complementary investigations.

Two LG− patients had had a histological confirmation of lymphoid neoplasm: one DLBCL was diagnosed on node biopsy (BLI negative) and one high-grade follicular lymphoma (FL) was diagnosed on skin biopsy (BLI positive).

Hematological and oncogeriatric evaluations were requested in, respectively, 25% and 31% of cases in the subgroup with positive BLI vs. 5.5% and 11% in the subgroup with negative BLI, resulting in a significant difference between the two groups (*p* = 0.02 and *p* = 0.04, respectively).

## 4. Discussion

This work is original because it reflects real-life BLI prescribing practices for older adults hospitalized in geriatric units. We also provided details on the most profitable BLI prescription for better diagnostic management of patients.

The study population was polypathological (67% of the population had an ACCI ≥ 6), and frequently cognitively impaired (mean MMSE score of 18.7/30) and dependent (only 38% of the population had an ADL score ≥ 5).

### 4.1. Criteria Appearing to Be Profitable for BLI Prescription

This paper shows that lymphocytosis remains a valid indication for the prescription of BLI in case of lymphoid neoplasm suspicion, especially if it is significant (i.e., at a rate ≥ 5 G/L). Our results suggest a high profitability in this indication, with a likelihood of detecting a lymphoid neoplasm of more than 60% (mainly CLL but also a significant number of MZL). However, because of the modest size of the LG+, this result should be considered with caution. In addition, a higher mean lymphocyte count was significantly associated with positive BLI in the LG− (*p* < 0.01). Even if lymphocytosis is not greater than 5 G/L, if it is found to be in the upper limit of the norm, it is a relevant factor in the decision to prescribe a BLI.

BLI was more frequently prescribed in isolated anemia (10.5%) than in isolated thrombocytopenia (7.7%). However, anemia was not significantly associated with BLI positivity in the LG−. This could be explained by the frequently multifactorial nature of anemia in older adults. This result raises the question of whether the value of BLI should be evaluated in the event of anemia, when the first-line etiological assessment turns out to be negative. A platelet count close to the lower bound of the norm was associated with more profitable BLI results in the LG− (*p* < 0.01). However, despite a trend (*p* = 0.06), real thrombocytopenia (<150 G/L) was not associated with this gain in profitability, probably due to an insufficient number of included patients. According to the literature, thrombocytopenia is common in lymphoid neoplasm, but not specific [1,12]. A trend toward a low platelet count, therefore, could appear to be a potentially relevant indication for BLI.

We can underline the high rate of BLI prescription in a context of monocytosis (9.1% of prescriptions). In our sample, 44 patients from the LG− (30%) had monocytosis, but no significant association was shown between this criterion (monocytosis) and positive BLI in LG−. Monocytosis can be found in cases of lymphoid neoplasm, but with no specificity for the latter. Tadmor et al., showed that 18.7% of patients suffering from DLBCL had monocytosis at the time of diagnosis and that its presence seemed to suggest a poor prognosis for hematologic malignancy [13]. However, performing BLI with this isolated criterion (monocytosis) does not seem justified.

Clinically, general weakness was the most common indication for BLI (19.6%) in the LG−, while it was not profitable for BLI positivity (*p* = 0.9). This could be explained by the fact that in OPs, general weakness is frequent, unspecific, rarely isolated, and sometimes multifactorial [14].

The presence of lymphadenopathy(ies) was the second most frequent indication for BLI prescription (11%) and was significantly associated with positive BLI. This seems consistent with the typical presentation of the main lymphoid neoplasm in which lymphadenopathy is often described [1]. In a study by Nixon et al., lymphoid neoplasm was diagnosed in 52% of 126 patients with lymphadenopathy of undetermined origin [15]. We did not find a study analyzing the profitability of BLI in the presence of lymphadenopathy.

Though the profitability of BLI in a context of splenomegaly was high in LG− (*p* = 0.02), splenomegaly was not a frequent indication for BLI (2%). lymphoid neoplasms are a frequent cause of unexplained splenomegaly in patients who have a histological analysis following a diagnostic splenectomy [16]. Moreover, in a study that included 75 patients suffering from lymphoid neoplasm and whose clinical presentation included a predominant splenomegaly, it was shown that it was largely small B-cell lymphoid neoplasm, with in particular 24% of splenic MZL [17].

Two OPs had had a BLI for suspected cutaneous lymphoma, and both cases obtained a negative result. The search for a circulating phase in epidermotropic cutaneous T-cell lymphoid neoplasm or T-cell lymphoma (CTCL) appears to be of additional interest. CTCL classically includes either mycosis fungoides, less aggressive and primarily cutaneous at the start, or Sezary syndrome, which is rarer, more aggressive, and often with an immediate circulating blood form [18]. Therefore, in addition to the search for circulating Sezary cells, the search for a T-cell clone in the circulating blood during the CTCL diagnosis is integrated into the stratification of these conditions, and its presence appears to be deleterious [19]. Regardless, the search for a circulating T-cell lymphoid neoplasm only applies once the histological diagnosis is made and does not seem to be validated in current practice and as a first-line. It can, however, be discussed (in association with a dermatological evaluation) in the presence of dermatological abnormalities such as infiltrated chronic erythematous plaques (mycosis fungoides) or erythroderma (Sezary syndrome) with no other obvious etiology.

### 4.2. Epidemiological Coherence of the Objectified Clonal Population Subtypes

In our study, the details of the cases with positive BLI showed a predominance of small B-cell lymphoid neoplasms, which is consistent with the literature [20]. Small B-cell lymphoid neoplasm is the lymphoid neoplasm type that circulates most in the blood [21]. Among these small-cell lymphoid neoplasms, excluding CLL, splenic MZL also has a high frequency of blood circulation [22]. These data are consistent with our sample in which MZL tops the malignant hemopathies diagnosed in the event of positive BLI in all groups combined (43%). In second position, there was a set of malignant hemopathies including MBL, CLL, and SLL. Although they have clinical and biological differences, these conditions have a common immunophenotyping with a Matutes score ≥ 4. The indications for BLI in this group of hemopathies are well defined, though, with a possibly underestimated incidence of MBL in older adults due to the paucity of warning signs.

For FL, we identified few positive BLIs (around 5%), yet it is relatively common small B-cell lymphoid neoplasm. This may in part be explained by a younger median age of onset, around 65 years in men [23].

Positive BLI was not found in association with mantle cell lymphoma (MCL). Although MCL has a fairly common circulating blood phase compared to other lymphoid neoplasms, its incidence is lower [23,24].

In our sample, only one patient had histologically confirmed DLBCL and no circulating blood phase was found, which was surprising since epidemiological data report a high preponderance of DLBCL among lymphomas. However, DLBCL has poor blood circulation due to its large B cells, which may explain its poor representation in our study [1,21]. In addition, it is possible that OPs likely to have DLBCL did not have sufficiently preserved general health to consider histological analysis.

### 4.3. Hematological Characteristics and Geriatric Specificities of the Study Population

The population studied here mainly showed low-grade mature B-cell lymphoid neoplasm, which are generally non-aggressive. The slow progression of most of these mature B-cell lymphoid neoplasms (excluding mantle cell lymphoma) can justify, especially in OPs with comorbidities, a reinforced surveillance strategy without curative treatment as a first step [25,26,27]. While simple treatments, such as the eradication of Helicobacter pylori in mucosa-associated lymphoid tissue (a subtype of extranodal MZL), are easy to implement, other strategies (splenectomy in splenic MZL, radiotherapy, immunotherapy, etc.) require more a careful patient assessment [25].

It is estimated that approximately 30% of people with DLBCL are at least 75 years of age, yet therapeutic trials for DLBCL rarely include octogenarians [28]. The few studies that do include Merli et al., Olivieri et al., and Spina et al., which focus on dependence in geriatric criteria [29,30,31]. There is also the work of Zeremski et al., focusing on chemotherapy in DLBCL in a population with a median age of 79 years, but with ACCI ≥ 6 in only 23% of individuals [32].

It is interesting to note that the geriatric assessment made it possible to reduce the chemotherapy protocols according to the degree of frailty, with relatively good therapeutic results [33].

Among the three OPs with histological confirmation of lymphoid neoplasm, the one DLBCL patient was frail and had a major neurocognitive disorder and an ADL score of 3/6. This patient received pre-phase treatment with vincristine, but then deteriorated until death. The other two patients, one with high grade FL and one with MZL, were more “robust” (ACCI < 6 and ADL score > 5) and obtained complete response with specific treatment (rituximab–cyclophosphamide–doxorubicin–vincristine–prednisone for the OP with high grade FL or rituximab alone for the OP with MZL).

Thus, when a blood lymphocyte clone is identified in BLI, excluding CLL, and there is an evocative clinical and possibly paraclinical picture, lymphoid neoplasm can be considered with virtual certainty. Histological confirmation would then be “reserved” for OPs who may benefit most from therapeutic management. Unfortunately, the most common lymphoma, DLBCL, is often aggressive and diagnosis is rarely helped by BLI, which tends to be negative.

Hematological and oncogeriatric evaluations were significantly more frequently requested following positive BLI, illustrating a desire to implement appropriate multidisciplinary management, highlighting the importance of the BLI result. Again, this study reinforces the importance of early oncogeriatric assessments in order to avoid unnecessary invasive diagnostic testing with potentially no therapeutic outcome.

### 4.4. Limitations

Our study has some limitations. The first is linked to its retrospective nature and the potentially non-exhaustive description of the criteria that led the physician to prescribe BLI. However, the initial cause for prescribing BLI was almost always clearly mentioned in the medical record. Another limitation of this study is the lack of multivariate analysis due to the low number of included patients, particularly in the subgroup of LG− with positive BLI.

## 5. Conclusions

This work shows that small mature B-cell lymphoid neoplasms were most commonly diagnosed in the study population in the event of positive BLI findings. Unfortunately, the diagnostic process for DLBCL, the most common lymphoma type, does not appear to be helped by BLI.

The reasons for performing a BLI in the event of clinical suspicion of lymphoid neoplasm in the geriatric population are based on various clinical or biological criteria. The discovery of lymphadenopathy, splenomegaly, lymphocytosis, and a low platelet count provides profitable clinical and biological criteria that suggest the need for a subsequent BLI in current practice.

The patients included in this study had comorbidities, polypharmacy, low functional independence, and cognitive disorders. The diagnostic and therapeutic approach is more complex for this type of patient; hence, the desire to strengthen the set of diagnostic arguments by first using techniques such as BLI, which are admittedly indirect but noninvasive. In the event of positive BLI, the combined contribution of the oncogeriatrician and hematologist is more often required, highlighting the need for appropriate and multidisciplinary management in OP with lymphoid neoplasm.

## Figures and Tables

**Table 1:** Characteristics of patients in the lymphocytosis group (LG+).

Variable			Positive BLI for CLL N = 3 (37.5%)	Positive BLI for MZL N = 2 (25%)	Negative BLI N = 3 (37.5%)
			Mean ± SD (min–max) or N (%)	Mean ± SD (min–max) or N (%)	Mean ± DS (min–max) or N (%)
Demographic data	Age		86.3 ± 3.2 (84–90)	82.5 ± 3.5 (80–85)	86.3 ± 8.5 (80–96)
	Female		2 (66.7)	2 (100)	3 (100)
Medical data	General weakness		1 (33.3)	1 (50)	0 (0)
	Lymphadenopathy(ies)		0 (0)	0 (0)	0 (0)
	Hepatomegaly		0 (0)	0 (0)	0 (0)
	Splenomegaly		0 (0)	1 (50)	0 (0)
	Unexplained fever		0 (0)	1 (50)	0 (0)
	Sweats		0 (0)	1 (50)	0 (0)
Biological data	Lymphocyte count (G/L)		8.3 ± 4.1 (5.5–13)	17.2 ± 10.2 (10–24.3)	5.5 ± 0.5 (5–5.9)
	Hemoglobin (g/dL)	Mean	11.2 ± 0.5 (10.7–11.7)	9.6 ± 2.1 (8.1–11.1)	12.6 ± 1.7 (11.3–14.5)
		Anemia	2 (66.7)	2 (100%)	0 (0)
	Platelet count (G/L)	Mean	241 ± 127 (157–388)	118 ± 49 (83–153)	291 ± 84 (199–363)
		Thrombocytopenia	0 (0)	1 (50)	0 (0)
	Serum protein electrophoresis	Prescription rate	2 (66.7)	2 (100)	2 (66.7)
		Monoclonality	1 (33.3)	0 (0)	1 (33.3)
		Normal	1 (33.3)	2 (100)	1 (33.3)
Geriatric assessment data	Mini Mental State Examination score		23.3 ± 9 (13–30)	16.5 ± 13.4 (7–26)	17.7 ± 5.7 (13–24)
	Activity of Daily Living score		3.7 ± 1.6 (2.5–5.5)	4 ± 2.8 (2–6)	4.2 ± 1.5 (2.5–5.5)
	ACCI		5.7 ± 0.6 (5–6)	5.5 ± 0.7 (5–6)	5.7 ± 0.6 (5–6)
	Albumin rate (g/L)		28.7 ± 4.7 (25–34)	23.5 ± 7.8 (18–29)	30.7 ± 4 (26–33)
	Living in nursing home		0 (0)	1 (50)	0 (0)
	Polypharmacy (≥ 5 drugs)		3 (100)	1 (50)	2 (66.7)
	Independent ambulation		1 (33.3)	1 (50)	1 (33.3)
Complementary investigations	Imagery done		1 (33.3)	2 (100)	0 (0)
	Solid biopsy done		0 (0)	1 (50)	0 (0)
	Histological confirmation of LN		0 (0)	1 (50)	0 (0)
Follow-up data	Hematological evaluation		0 (0)	1 (50)	0 (0)
	Oncogeriatric evaluation		1 (33.3)	1 (50)	0 (0)

BLI: blood lymphocyte immunophenotyping, CLL: chronic B lymphocytic leukemia, MZL: marginal zone lymphoma, N: number, SD: standard deviation, min: minimal, max: maximal, G/L: Giga per liter, g/dL: grams per deciliter, ACCI: age-adjusted Charlson comorbidity index, g/L: grams per liter, LN: lymphoid neoplasm.

**Table 2:** Indications for blood lymphocyte immunophenotyping prescription in the absence of lymphocytosis (LG−).

Indications for Blood Lymphocyte Immunophenotyping		LG− N = 143
		N (%)
Clinical criteria	General weakness	28 (19.6)
	Lymphadenopathy(ies)	11 (7.7)
	Unexplained fever	2 (1.4)
	General weakness + lymphadenopathy(ies)	1 (0.7)
	General weakness + hepatomegaly	1 (0.7)
	General weakness + unexplained fever	1 (0.7)
	General weakness + sweats	1 (0.7)
	General weakness + lymphadenopathy(ies) + splenomegaly	2 (1.4)
	General weakness + lymphadenopathy(ies)+ hepatomegaly	1 (0.7)
	General weakness + lymphadenopathy(ies) + unexplained fever	1 (0.7)
	General weakness + unexplained fever + sweats + splenomegaly	1 (0.7)
	Lymphadenopathy(ies) + hepatomegaly + splenomegaly	1 (0.7)
	polyserositis	1 (0.7)
	Suspicion of digestive lymphoma	2 (1.4)
	Suspicion of cerebral lymphoma	3 (2.1)
	Suspicion of cutaneous lymphoma	2 (1.4)
	Follow-up to known lymphoma	1 (0.7)
	Lung lesion + lymphadenopathy(ies) + splenomegaly	1 (0.7)
	Deep infection	1 (0.7)
Biological criteria	Anemia	15 (10.5)
	Thrombocytopenia	11 (7.7)
	Anemia + thrombocytopenia	10 (7)
	Anemia + neutropenia	1 (0.7)
	Pancytopenia	5 (3.5)
	Polycythemia	3 (2.1)
	Thrombocytosis	1 (0.7)
	Monocytosis	13 (9.1)
	Hypereosinophilia	3 (2.1)
	Biological inflammatory syndrome	9 (6.3)
	Hypercalcemia	2 (1.4)
	Monoclonal gammapathy at immunoglobulin M	3 (2.1)
	Hypogammaglobulinemia	4 (2.8)
	Polyclonal hypergammaglobulinemia	1 (0.7)

LG−: Group with no significant lymphocytosis; N: number.

**Table 3:** Comparison of patients with no lymphocytosis (LG−) according to blood lymphocyte immunophenotyping result.

Variable				LG−		
				Positive BLI N = 16	Negative BLI N = 127	*p*
				Mean ± SD (min–max) or N (%)	Mean ± SD (min–max) or N (%)	
Demographic data	Age			87.5 ± 5.2 (79–97)	86.7 ± 5.8 (75–97)	0.6
	Male			9 (56.2)	67 (52.7)	0.8
Clinical data	History of LN			2 (12.5)	7 (5.5)	0.3
	General weakness			7 (44)	57 (45)	0.9
	Lymphadenopathy(ies)			7 (44)	25 (20)	0.05
	Hepatomegaly			3 (19)	7 (5.5)	0.08
	Splenomegaly			4 (25)	7 (5.5)	0.02
	Unexplained fever			2 (12.5)	6 (4.7)	0.2
	Sweats			1 (6)	3 (2.4)	0.4
	Pruritus			0 (0)	1 (0.8)	1
Biological data	Mean lymphocyte count (G/L)			2.1 ± 1.4 (0.3–3.9)	1.3 ± 0.65 (0.3–3.9)	<0.01
	Hemoglobin (g/dL)	Mean		10.9 ± 2.1 (8.2–14.4)	11 ± 2 (6–16.6)	0.9
		Anemia		11 (69)	76 (60)	0.6
		Polyglobulia		0 (0)	0 (0)	1
	Platelet count (G/L)	Mean		172 ± 80 (80–364)	276 ± 146 (7–893)	<0.01
		Thrombocytopenia		7 (44)	27 (21)	0.06
		Thrombocytosis		0 (0)	9 (7)	0.6
	Leukocyte count (G/L)	Mean		9.1 ± 4.7 (3.2–18.3)	9 ± 7.1 (1.8–59.3)	0.9
		Leucocytosis		4 (25)	32 (25)	1
		Leucopenia		3 (19)	12 (9)	0.4
		Eosinophils (G/L)	Mean	0.09 ± 0.09 (0–0.3)	0.17 ± 0.23 (0–2)	0.1
			High	0 (0)	3 (2.4)	1
		Monocytes (G/L)	Mean	0.65 ± 0.38 (0.12–1.34)	0.96 ± 1 (0.07–8.9)	0.22
			High	4 (25)	40 (31)	0.77
	CRP			65 ± 91 (3–323)	44 ± 53 (3–263)	0.2
	Serum protein electrophoresis	Prescription rate		14 (88)	104 (82)	0.7
		Monoclonality		5 (36)	14 (13)	0.1
		Hypogammaglobulinemia		2 (14)	7 (7)	0.3
		Normal		7 (50)	70 (67)	0.2
		Polyclonal hypergammaglobulinemia		0 (0)	12 (12)	0.3
Geriatric data	MMSE score	Mean		13.8 ± 5.6 (3–23)	19.2 ± 5.9 (6–30)	<0.01
		Missing data		12.5%	9%	
	Activity of daily living score			3.6 ± 1.3 (1–6)	3.9 ± 1.6 (0–6)	0.4
	Age-adjusted Charlson comorbidity index			7.4 ± 2.1 (5–11)	6.4 ± 1.7 (3–15)	0.03
	Albumin rate (g/L)			30.3 ± 3.5 (25–38)	27.2 ± 5 (14–41)	0.02
	Living in nursing home			2 (12.5)	19 (15)	0.8
	Polypharmacy			12 (75)	100 (78)	0.7
	Independent ambulation			9 (56)	68 (54)	0.8
	LMI allowing to claim resuscitation			3 (19)	36 (28)	0.5
Complementary investigations	Thoracic abdominal and pelvic computed tomography			7 (44)	43 (34)	0.4
	Positron emission tomography			2 (12.5)	7 (5.5)	0.3
	Myelogram			2 (12.5)	18 (14)	1
	Bone marrow biopsy			1 (6)	1 (0.8)	0.2
	Node biopsy			1 (6)	1 (0.8)	0.2
	Extranodal biopsy			1 (8)	0 (0)	0.1
	Histological confirmation of LN			1 (6)	1 (0.8)	0.2
Follow-up data	Hematological evaluation			4 (25)	7 (5.5)	0.02
	Oncogeriatric evaluation			5 (31)	14 (11)	0.04

LG−: group with no lymphocytosis, BLI: blood lymphocyte immunophenotyping, N: number, SD: standard deviation, min: minimal, max: maximal, LN: lymphoid neoplasms, G/L: Giga per liter, g/dL: grams per deciliter, CRP: C-reactive protein, MMSE: Mini Mental State Examination, g/L: grams per liter, LMI: level of medical intervention.

## Data Availability

The data presented in this study are available on request from the corresponding author. The data are not publicly available.
